# Seasonal variations in acute diverticular disease hospitalisations in New Zealand

**DOI:** 10.1007/s00384-023-04338-4

**Published:** 2023-02-16

**Authors:** Chris Varghese, Zhenqiang Wu, Ian P. Bissett, Martin J. Connolly, Joanna B. Broad

**Affiliations:** 1 Department of Geriatric Medicine, The University of Auckland, PO Box 93 503, 124 Shakespeare Road, Takapuna, Auckland, New Zealand; 2 Department of Surgery, The University of Auckland, Auckland, New Zealand; 3 School of Population Health, The University of Auckland, Auckland, New Zealand; 4 Waitematā District Health Board, Auckland, Auckland, New Zealand

**Keywords:** Diverticular disease, Trends, Seasonal variation, Vitamin D

## Abstract

**Purpose:**

Seasonal variation of acute diverticular disease is variably reported in observational studies. This study aimed to describe seasonal variation of acute diverticular disease hospital admissions in New Zealand.

**Methods:**

A time series analysis of national diverticular disease hospitalisations from 2000 to 2015 was conducted among adults aged 30 years or over. Monthly counts of acute hospitalisations’ primary diagnosis of diverticular disease were decomposed using Census X-11 times series methods. A combined test for the presence of identifiable seasonality was used to determine if overall seasonality was present; thereafter, annual seasonal amplitude was calculated. The mean seasonal amplitude of demographic groups was compared by analysis of variance.

**Results:**

Over the 16-year period, 35,582 hospital admissions with acute diverticular disease were included. Seasonality in monthly acute diverticular disease admissions was identified. The mean monthly seasonal component of acute diverticular disease admissions peaked in early-autumn (March) and troughed in early-spring (September). The mean annual seasonal amplitude was 23%, suggesting on average 23% higher acute diverticular disease hospitalisations during early-autumn (March) than in early-spring (September). The results were similar in sensitivity analyses that employed different definitions of diverticular disease. Seasonal variation was less pronounced in patients aged over 80 (*p* = 0.002). Seasonal variation was significantly greater among Māori than Europeans (*p* < 0.001) and in more southern regions (*p* < 0.001). However, seasonal variations were not significantly different by gender.

**Conclusions:**

Acute diverticular disease admissions in New Zealand exhibit seasonal variation with a peak in Autumn (March) and a trough in Spring (September). Significant seasonal variations are associated with ethnicity, age, and region, but not with gender.

**Supplementary Information:**

The online version contains supplementary material available at 10.1007/s00384-023-04338-4.

## Introduction

Diverticulosis describes outpouchings of the colonic wall that are typically asymptomatic [1]. Diverticulosis is common in Western countries [2]; the prevalence varies with age, increasing from < 10% in adults under 40 years to 65% in those aged 70 years and above [1, 3]. Around 10–20% of those with diverticula manifest acute clinical complications, and this is classified as diverticular disease (e.g. diverticulitis or diverticular haemorrhage) [1], which are associated with a significantly higher burden of healthcare cost [4–6]. In the USA, over 200,000 hospital admissions for primary diagnosis of diverticulitis without haemorrhage occurred in 2012 at a cost of 2.1 billion US dollars [7].

Despite diverticular disease being one of the most common, and growing gastrointestinal conditions, especially in older people, its pathogenesis is poorly understood. Research into risk factors may yield clues towards better understanding its etiology and thus inform prevention and treatment. Identified risk factors include age [3, 8], obesity [9], lower physical activity [10], low dietary fibre [11], connective tissue disorders [12], and genetic factors [13]. Seasonality, ultraviolet light exposure, and vitamin D status have been investigated in several observational studies of diverticular disease [14–18]; however, inconsistent results make it difficult to assess the relationship [19]. A post-hoc analysis of a randomised controlled trial data indicated that vitamin D supplementation in a general population with low vitamin D levels may reduce the risk of diverticular disease hospitalisations compared to placebo [20]. However, it remains unresolved if diverticular disease presentations have seasonal patterns, in particular whether exposure to sunshine during summer months may be associated with lower diverticular disease occurrence, or winter months with higher occurrence.

Few studies offer evidence on seasonality of diverticular disease, with mixed findings and only one presents data from the southern hemisphere. In New Zealand, Europeans are the predominant ethnic group, and Māori are the indigenous population. In 2018 Census, the population comprised 70% European, 17% Māori, 15% Asian, 8% Pacific Peoples, and 2% Middle Eastern, Latin American, and African peoples [21], though these proportions have varied over the time period of the study. The population was 3.86 million in 2000, rising to 4.61 million in 2015, and over 5 million in 2022 [22]. New Zealand has a public health system which provides free access to hospital care with a small private sector that provides mainly non-acute care. The New Zealand Ministry of Health’s national collections record mortality, hospital discharges, and health services, including both publicly and privately provided care (if publicly funded) [23]. In this study, we therefore aimed to perform a time series analysis of national hospitalisation data in New Zealand, to investigate seasonal variations in acute diverticular disease hospital admissions.

## Materials and methods

This was a time series analysis of national hospitalisation records from January 2000 to December 2015 in New Zealand. Data were extracted from the national minimum dataset—a collection of public and private hospital discharge information [24]. For each hospital admission, the date of admission, primary diagnosis code, region of treatment agency, admission type (acute or non-acute), domicile code, and length of stay were extracted, together with demographic data (year of birth, gender and self-identified prioritised ethnicity). Patients who were aged 30 years or over at acute diverticular disease hospital admission and were residents of New Zealand were included in the study. Acute diverticular disease hospital admissions were defined as acute hospitalisation (admission type = “acute” which included emergency department presentations of over 3 h and arranged admissions where time to admission is under 24 h from referral) with a diverticular disease discharge diagnosis (ICD-10 codes “K57.0-K57.9”, diverticular disease of the intestine) as the primary discharge diagnosis [25]. In New Zealand, the primary diagnosis record for each hospital admission is mandatory, but not the secondary diagnoses. Therefore, an admission can be reliably attributed to diverticular disease, but it is not possible to confidently ascribe from ICD-10-coded data to different subgroups, for example, acute diverticulitis, diverticular perforation/abscess, or diverticular bleed [26, 27]. Those with region missing, non-New Zealand residents, or with length of stay more than 60 days, were excluded. For patients with more than one hospital admission in a day, second or subsequent hospital admissions were excluded. In order to account for any effects from varied definitions of acute diverticular disease, we adapted two further definitions from previous studies and conducted two sensitivity analyses. In the first sensitivity analysis, we excluded patients with a diagnosis of small bowel diverticula (ICD-10 diagnosis codes K57.0 or K57.1) [27], and in the second sensitivity analysis, we included only patients with acute diverticulitis (ICD-10 diagnosis codes of K57.2, K57.32, K57.33, or K57.9) [14, 16, 28].

Monthly average acute diverticular disease counts were calculated, adjusting for lengths of different months. Unlike some studies, we did not express the observed counts over the total hospitalisations as we consider that unjustified and would introduce bias. To assess the monthly seasonality of acute diverticular disease counts, we used the X-11 method from the United States Census Bureau, which is a widely used seasonal adjustment method [29]. Monthly acute diverticular disease counts were decomposed to (1) trend component over the duration of the study, (2) seasonal component, and (3) irregular component. The seasonal component was extracted for each month to describe the volume of acute diverticular admissions per month adjusted for the overall trend and irregular component.

Seasonality of monthly acute diverticular disease counts were determined by the stable seasonality F-test (at 0.001 significant level), moving seasonality *F*-test (at 0.05 significance level), and Kruskal–Wallis Chi-squared test (at 0.001 significant level), and the combined test for identifiable seasonality that assessed all three tests [30]. If seasonality was identified, the mean peak month, mean trough month, and annual seasonal amplitude with 95% confidence intervals (CIs) were calculated. To calculate the annual seasonal amplitude, the difference between peak and trough monthly seasonal components of each year was divided by the mean monthly seasonal component of that year to calculate the seasonal amplitude. Seasonal amplitudes were presented as percentages and can be interpreted as the amount of monthly variation of acute diverticular disease admissions. CIs were calculated based on the *t*-distribution. One-way analysis of variance (ANOVA) was used to compare mean seasonal amplitudes between subgroups (gender, age, ethnicity, and region) when the distributions were parametric; otherwise, the Kruskal–Wallis test was used. All statistical analyses were performed in SAS version 9.4 (SAS Institute, Cary, NC, USA). A two-sided *p* < 0.05 was considered statistically significant.

## Results

A total of 37,197 acute hospital admissions for diverticular disease occurred between January 2000 and December 2015. Of those, 183 admissions were excluded due to age less than 30 years at time of admission; a further 1432 were excluded due to missing region (*n* = 216), not a resident of New Zealand (*n* = 910), length of stay more than 60 days (*n* = 29), or subsequent same-day admissions (*n* = 277). Thus, 35,582 patients admitted with acute diverticular disease at hospital discharge were included.

**Fig. 1:**
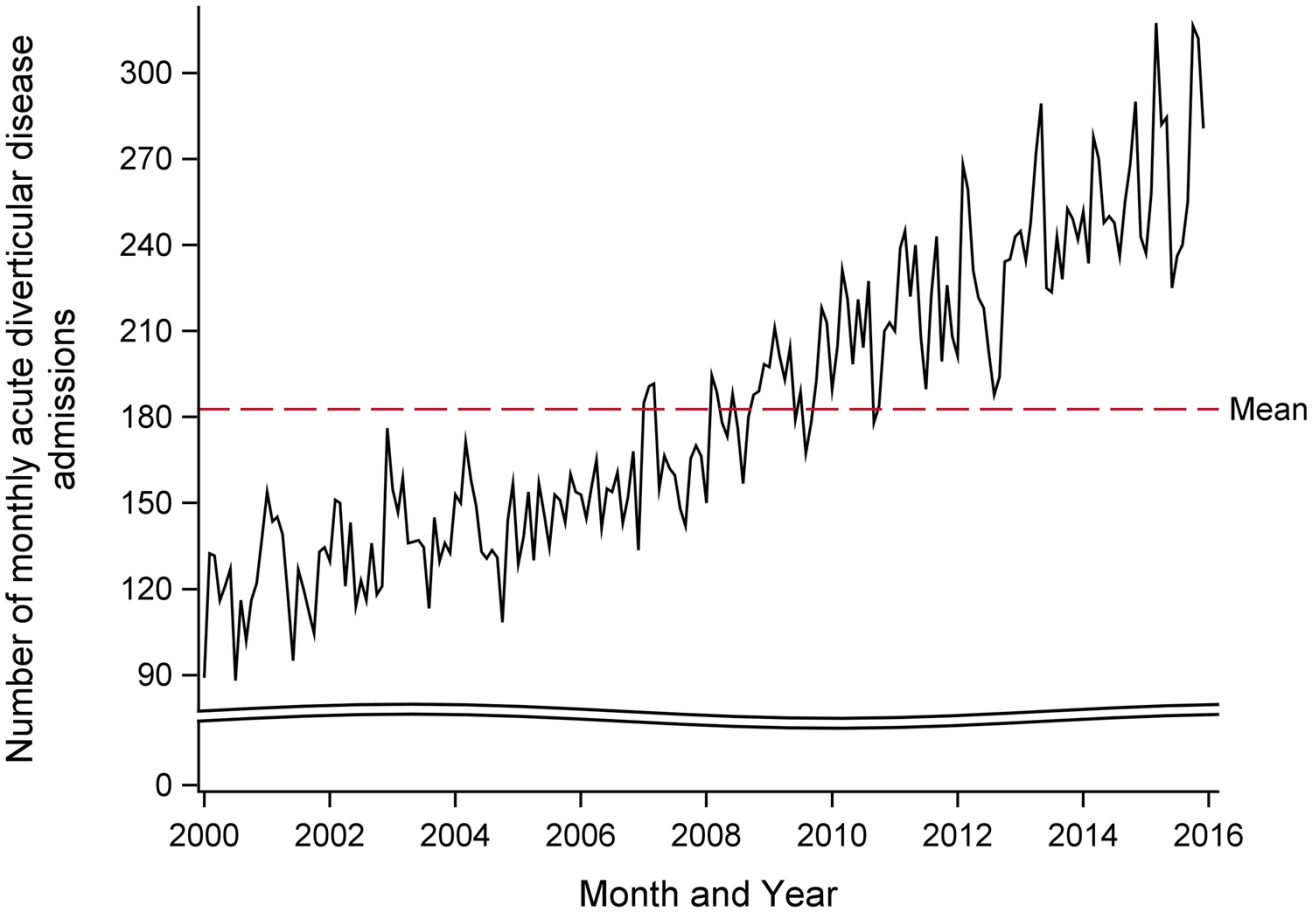
Time series of monthly number of acute diverticular disease admissions in New Zealand adults over 30 years of age, 2000–2015. Red line represents the mean monthly number of diverticular disease admissions over the study period

The mean (SD) age of the patients at hospital admission was 65.2 (15.0) years. Most of the acute diverticular disease admissions were of European/other ethnicity (31,159, 87.6%), with much lower numbers among Māori (2600, 7.3%), Pacific Peoples (1139, 3.2%), and Asian populations (684, 1.9%). The characteristics of the hospital admissions are summarised in Table 1. The average, unadjusted, monthly count of acute diverticular disease admissions over the 16-year study period are shown in Fig. S1. Figure 1 shows monthly acute diverticular disease counts from January 2000 to December 2015. The mean monthly diverticular disease count was 182.7 (SD 51.3), ranging from 88.1 to 317.4.

**Table 1:** Characteristics of acute diverticular disease hospital admissions in New Zealand adults aged 30 years of age and over between 2000 and 2015

**Characteristic**	**All acute diverticular disease admissions (*n*** **= 35,582)**
Age (year), mean (SD)	65.2 (15.0)
Age (year), *n* (%)	
30–39	1524 (4.3)
40–49	4566 (12.8)
50–59	7150 (20.1)
60–69	7509 (21.1)
70–79	7538 (21.2)
≥ 80	7295 (20.5)
Gender, *n* (%)	
Women	19,667 (55.3)
Men	15,915 (44.7)
Ethnicity, *n* (%)	
European/others	31,159 (87.6)
Māori	2600 (7.3)
Pacific Peoples	1139 (3.2)
Asian	684 (1.9)
Region, *n* (%)	
Northern	12,194 (34.3)
Midland	7198 (20.2)
Central	8380 (23.6)
Southern	7810 (21.9)

**Fig. 2:**
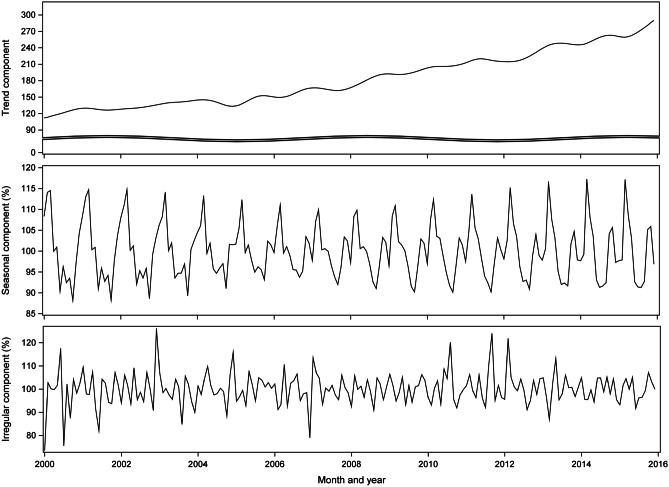
Time series decomposition of monthly number of acute diverticular disease admissions in New Zealand adults over 30 years of age between 2000 and 2015

Monthly acute diverticular disease occurrence was decomposed to trend, seasonal, and irregular components (Fig. 2). Although there are fluctuations in the long-term trend over the study period, there appears to be a general increasing trend of acute diverticular disease presentations. The seasonal component indicates that the seasonal amplitude decreased steadily from 2000 to 2008 and increased thereafter.

**Fig. 3:**
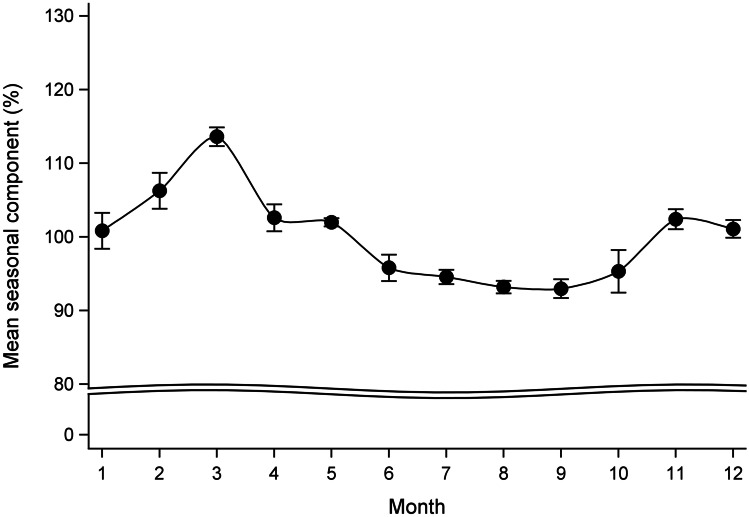
Mean seasonal component (95% confidence interval) of monthly number of acute diverticular disease admissions in New Zealand adults over 30 years of age between 2000 and 2015

The combined seasonality test clearly identified seasonality in monthly acute diverticular disease counts. The mean monthly seasonal component of acute diverticular disease occurrence over the study period peaked in the month of March (early-autumn in the southern hemisphere) and troughed in the month of September (early-spring) (Fig. 3). The mean annual seasonal amplitude was 22.9% (95%CI = 21.2–24.7), suggesting an annual mean of 22.9% additional acute diverticular disease hospitalisations during the peak month relative to the trough month. After excluding 592 patients with a primary diagnosis of small bowel diverticula (ICD-10 codes of K57.0 or K57.1) in a sensitivity analysis (*n* = 34,990), we found significant seasonality in monthly acute diverticular disease counts, peaked seasonal component in March and troughed in August (Fig. S2), and similar annual seasonal amplitude of 23.7 (95%CI = 21.9–25.5). Similar results were found in acute diverticulitis patients (89% of them without bleeding), with peaked seasonal component in March and troughed in August (Fig. S3), and similar annual seasonal amplitude of 26.5 (95%CI = 25.1–28.0).

Table 2 highlights comparisons for the seasonal amplitude of acute diverticular disease by gender, age, ethnicity, and region. Seasonal variation was most pronounced in those aged between 50 and 59 (*p* = 0.002). Māori had significantly higher seasonal variations than Europeans (*p* < 0.001). Northern regions had significantly lower seasonality than southern regions of New Zealand (*p* < 0.001). No significant difference in seasonal variation was observed by gender.

**Table 2:** Annual seasonal amplitude of monthly acute diverticular disease in New Zealand adults over 30 years of age between 2000 and 2015

	**Peak/trough month**	**Mean (CI) seasonal amplitude (%)**	**ANOVA** ***p*** **value**
All admissions	March/September	22.9 (21.2–24.7)	
Gender			0.56
Women	March/August	26.3 (24.7–28.0)	
Men	March/July	25.0 (20.4–29.6)	
Age			0.002
30–39	NA		
40–49	August/June	30.4 (25.7–35.1)	
50–59	February/September	40.9 (35.2–46.7)	
60–69	March/July	36.9 (32.5–41.3)	
70–79	December/August	33.5 (29.4–37.6)	
≥ 80	March/December	30.5 (27.8–33.3)	
Ethnicity			< 0.001
European/others	March/August	23.9 (21.4–26.3)	
Maori	March/June	54.2 (52.7–55.7)	
Pacific Peoples	NA		
Asian	NA		
Region			< 0.001
Northern	April/June	26.2 (23.1–29.3)	
Midland	November/July	30.6 (28.1–33.1)	
Central	March/October	35.6 (33.0–38.3)	
Southern	March/October	33.9 (29.7–38.1)	

*NA* not available due to low numbers, *CI* 95% confidence interval

## Discussion

The time series analysis of national hospitalisation data between 2000 and 2015 in New Zealand adults aged 30 years or over found a seasonal variation in acute admission and primary diagnosis of diverticular disease counts, with a peak in early-autumn (March) and trough in early-spring (September). The diverticular disease count on average was nearly a quarter higher in the peak month compared to the trough month. Seasonal variations were similar by gender, but varied by age, were markedly higher among Māori, and those living in more southern latitudes.

Previous observational studies of the seasonality of diverticular disease have been analysed and reported variably. On the one hand, are those which used an overall objective statistical test for seasonality. For example, a study from the USA included 412,163 general surgery-related emergency admissions with a first diagnosis of diverticulitis (ICD-9 codes of 562.11 or 562.13) from the national inpatient sample (NIS) database over an 8-year period (2004–2011) [28]; it reported significant seasonality on monthly diverticulitis counts (*p* < 0.001) using X-11 decomposition methods, with unchanged results after adjusting for estimated monthly US population (*p* < 0.001). This study observed a summer (July/August) peak and winter trough (February) of monthly diverticulitis admissions’ count and rate (per 100,000 NIS admissions or per 100,000,000 US population). A retrospective cohort analysis of 374,123 non-elective diverticulitis admissions (primary diagnosis, ICD-9 codes of 562.11 or 562.13) between 1997 and 2005 [14], also from the NIS database and using the same methods, revealed similar significant seasonality of monthly non-elective diverticulitis count (*p* < 0.001) and rates (per 100,000 NHI admissions, *p* < 0.01), and with the observed peak in summer months (July/August) and trough in winter months (January/February). A third study examined 18,672 acute primary diagnosis of diverticulitis (ICD-9 codes of 562.11, 562.13 or ICD-10 codes of K57.20, K57.21, K57.22, K57.23, K57.32, K57.33, K57.90, K57.91, K57.92, K57.93) from more than five million hospitalisations in both hemispheres and found significant sinusoidal pattern (from a cosinor model) of monthly acute diverticulitis rates (per 10,000 admissions, *p* < 0.025) that peaked in summer months across three countries (Australia, February; the UK, July–August; and the USA, July) [16]. This study also found winter months were associated with increased odds of hospital admission incidence for diverticulitis in the UK (August, OR = 1.22, 95%CI = 1.10–1.28) and the USA (August, OR = 1.24, 95%CI = 1.08–1.44) though not for Australia. These three studies, which used the comparable definition of diverticular disease with our sensitivity analysis, and the X-11-ARIMA or cosinor models, identified seasonality with peak occurring consistently in the summer months and trough in the winter months.

On the other hand, a small number of studies reported only the observed monthly or quarterly seasonal variation in diverticular disease without adjusting for any overall trend [15, 17, 18, 31]. The findings of these studies are less consistent, but most show summer peaks and winter troughs. For example, a Danish study of emergency admissions for complicated colonic diverticulitis, which included 44,160 acute admissions between 2002 and 2012, observed an autumn peak (September, October, and November) [18]. Similarly, an Italian study that included 29,428 hospital admissions of acute diverticulitis observed a September (early-autumn) peak of admissions [17]. Another US study, also based on NIS data, reported a lower rate in winter months (645 per 100,000 admissions) compared with summer (748 per 100,000 admissions) [15]. Two studies, however, had contrasting results. Yousaf et al. observed a summer peak and autumn trough of diverticulitis admissions using data from a hospital in the UK between 2013 and 2016 [32]. However, this study had small numbers of diverticulitis hospitalisations over the study period (*n* = 1101). In addition, a retrospective cohort study enrolled 133,875 patients with diverticular disease from 1988 to 2002 reported no evidence of seasonality and did not provide monthly or quarterly diverticular disease counts or rates [33].

Seasonal variation is therefore a likely factor in the occurrence of acute diverticular disease. The relationship between seasonality in acute diverticular disease hospitalisations and the etiology diverticulitis, however, remains unclear [34]. Further exploration of seasonally varying risk factors is therefore warranted. For example, the association of low vitamin D status and occurrence of diverticular disease has been investigated in previous literature but remains unresolved [15, 16, 19, 20, 35]. Vitamin D status is highly influenced by ultraviolet light exposure [36], and through the calendar year, in New Zealand, the prevalence of vitamin D deficiency is lowest from November through to March and then rises to higher levels between August and October [37]. Post-hoc analysis of randomised controlled trial data showed that vitamin D supplementation did not influence hospitalisation rates in diverticular disease in general; however, there may be benefit for those with vitamin D deficiency [20]. Our findings of a March peak and September trough of acute diverticular disease admissions in comparison to much lower proportions of adults with low vitamin D levels observed in March (< 1%) than in September (> 15%) in New Zealand [38] suggest that vitamin D influences diverticular disease hospitalisations, little if at all. Other seasonal behavioural factors such as diet and exercise may influence diverticular disease hospitalisations, potentially confounding any association with vitamin D. Further research is required to resolve the contribution of seasonally varying risk factors such as physical activity and seasonal patterns in the microbiome on diverticular disease hospitalisations. To investigate these associations, we suggest the use of existing prospective studies and a meta-analysis of randomised controlled trials of supplementary vitamin D.

The seasonality apparent from our study does not readily relate to the main recognised risk factors; increased age and genetics/family history do not vary with the season, physical activity (likely to be higher during summer months, so if anything, would be protective), leaving obesity, and diet. For most in New Zealand, summer is the time of eating fresh salads and seasonal fruits, together with increased outdoor physical activity accompanied by greater vitamin D production. Hypothetically, red meat consumption may increase over summer months when barbecues are popular, and perhaps the impact of that could accumulate and carry through into autumn. It is possible that seasonal changes in the microbiome or in alcohol consumption could be relevant, but the evidence for these as risk factors is sparse.

The strengths of this study include a national hospitalisation database with 16 years of data, which improves reliability and accuracy of our findings, particularly for the southern hemisphere where seasonality data is sparse. We analysed acute diverticular disease counts (not rates) to avoid the influence of any seasonality of overall hospital admissions or the general population over this long study period. Moreover, the use of widely used seasonal adjustment methods (Census X-11) and a combined test to identify seasonality improves the internal validity of our seasonality findings. This study also has several limitations. Our reliance on ICD-10 codes is limited by the accuracy of coding, and it is not possible to confidently ascribe from ICD-10-coded data to different subgroups, for example, acute diverticulitis, diverticular perforation/abscess, or diverticular bleed [26]. Therefore, our seasonality findings cannot be really linked to the detailed subgroups of acute diverticular disease. Coding accuracy, appropriateness, and timeliness of the discharge diagnosis were not possible to validate. Despite our attempt to account for potential sources of seasonal variation by analysing seasonal amplitude for different demographic subgroups, data for other potentially causal factors such as seasonal dietary patterns, obesity, and physical activity were not collected or modelled in our study. Further, ethnicity-based variations may be explained or confounded by different age distributions between Māori and non-Māori with diverticular disease [27] and with regional distribution, with many Māori living in northern and central regions [39]. Overall, causal factors and sources of the seasonality of acute diverticular disease remain poorly explained.

## Conclusion

Acute diverticular disease admissions in New Zealand exhibit seasonal variations with a peak in early-autumn (March) and a trough in early-spring (September). Seasonal variations are significantly associated with ethnicity, age, and region, but not with gender. This study highlights how little is known about the causal risk factors of diverticular disease and the importance of using established cohort to develop and clarify knowledge in this area. In particular, further work is needed on seasonally varying risk factors such as diet, physical activity, and changes in the gut microbiota to identify and inform preventative strategies for diverticular disease.

### Supplementary Information

Below is the link to the electronic supplementary material.

Supplementary file1 (DOCX 179 KB)

## Data Availability

The data underlying this article will be shared upon reasonable request to the corresponding author.
