# Supplementary material for: Seasonal variations in acute diverticular disease hospitalisations in New Zealand
Source: Int J Colorectal Dis. 2023 Feb 16;38(1):46. doi: 10.1007/s00384-023-04338-4 (PMC9935723; doi:10.1007/s00384-023-04338-4)
Supplement: Supplementary file 1 — Supplementary file1 (DOCX 179 KB) [file 384_2023_4338_MOESM1_ESM.docx]

**Supplementary material**

**
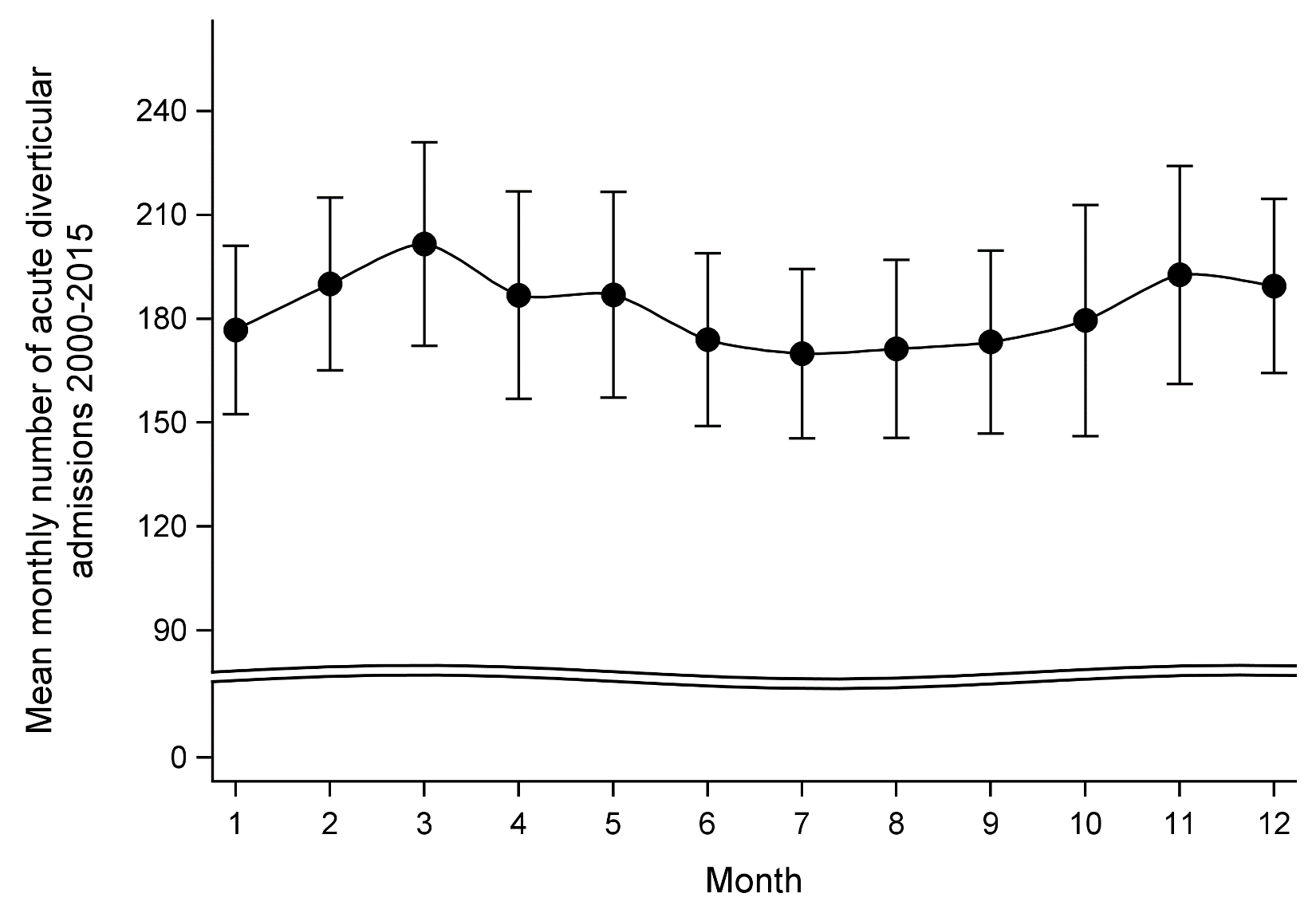
**

## Figure S1 Mean monthly number of acute diverticular disease admissions (95% confidence interval) in New Zealand adults over 30 years of age between 2000 and 2015.


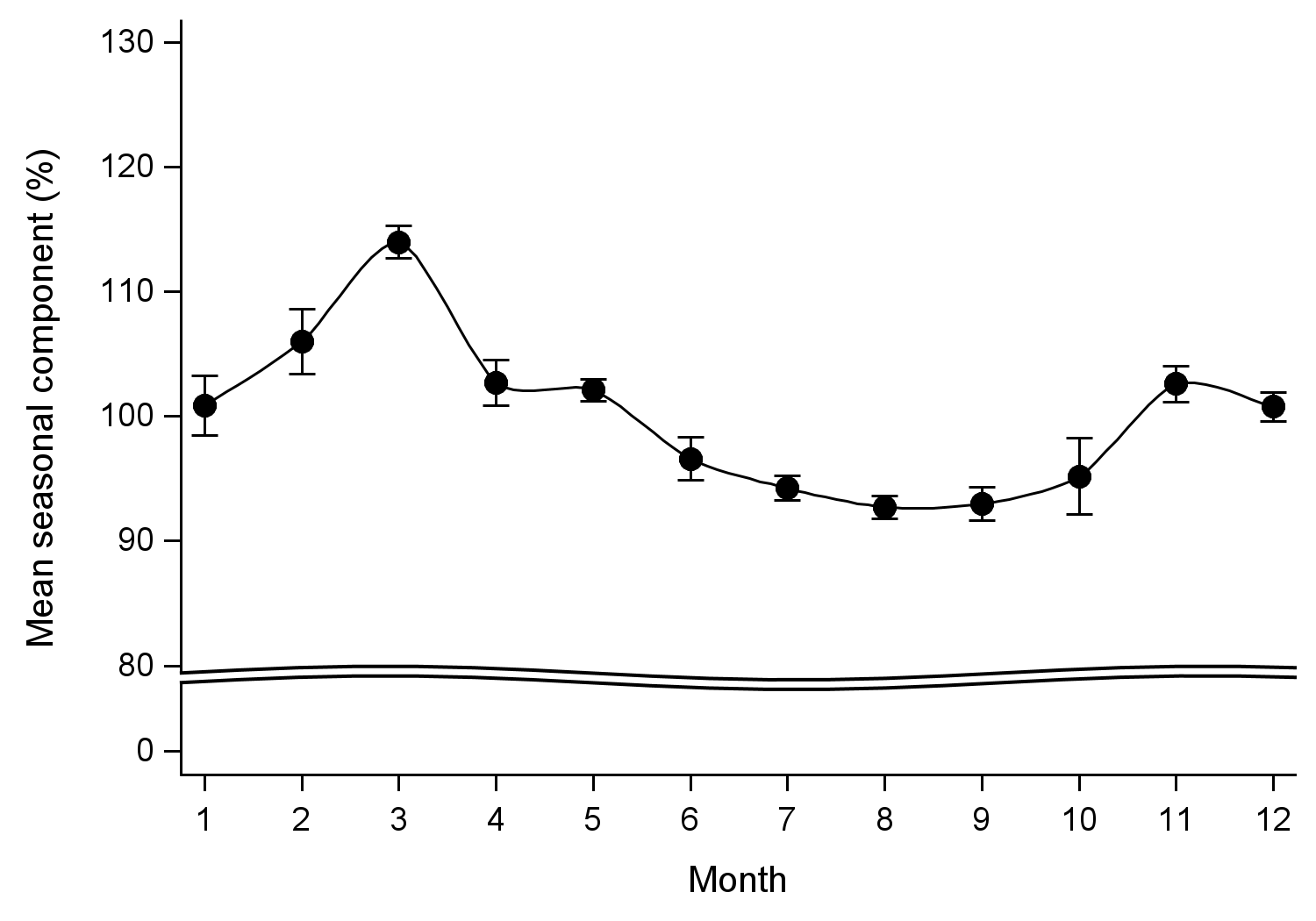


## Figure S2 Mean seasonal component (95% confidence interval) of monthly acute diverticular disease admissions with ICD-10 codes from K57.2 to K57.9.


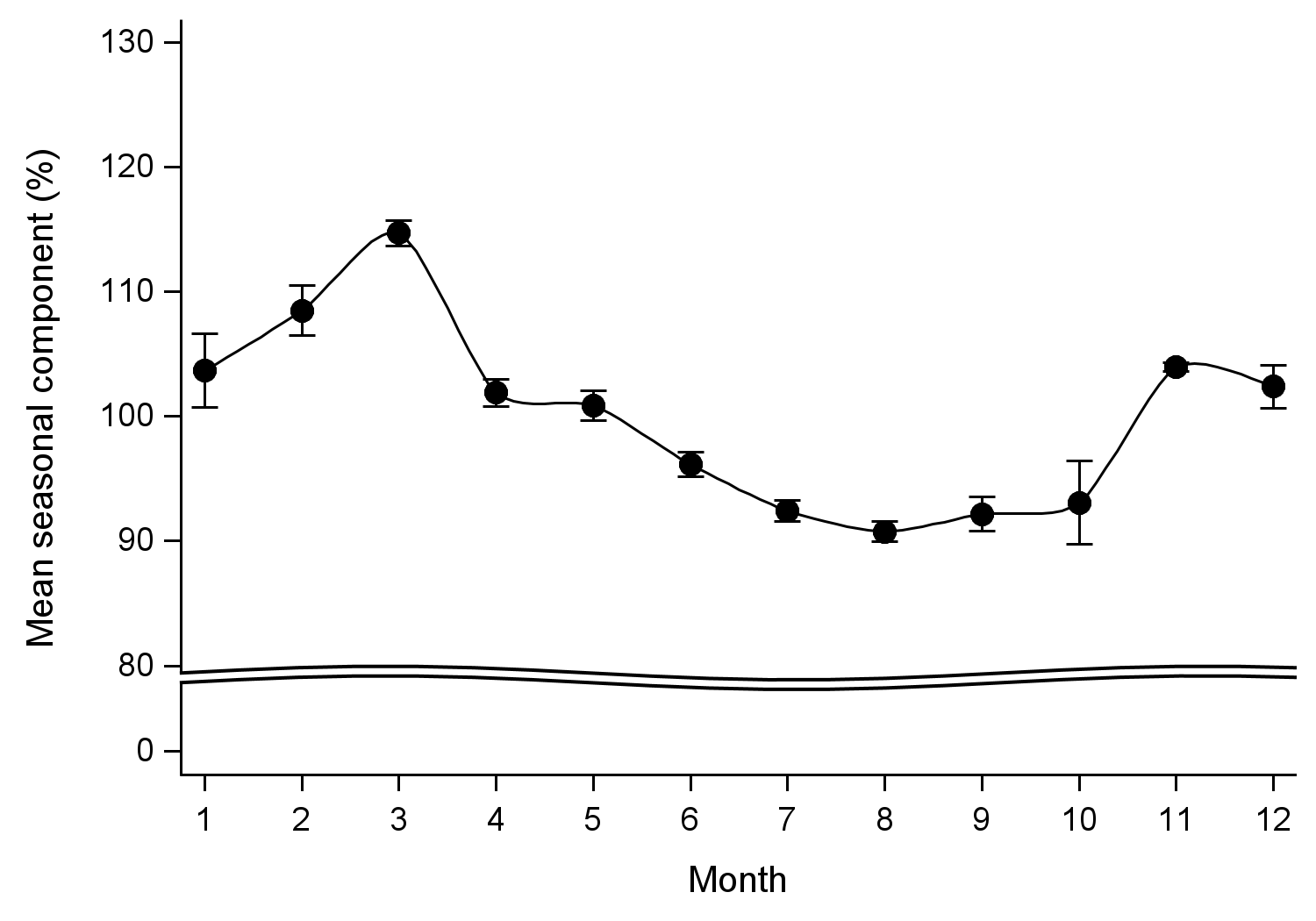


## Figure S3 Mean seasonal component (95% confidence interval) of monthly acute diverticular disease admissions with ICD-10 codes of K57.2, K57.32, K57.33, or K57.9.
